# Prediction of Dementia in Primary Care Patients

**DOI:** 10.1371/journal.pone.0016852

**Published:** 2011-02-18

**Authors:** Frank Jessen, Birgitt Wiese, Horst Bickel, Sandra Eiffländer-Gorfer, Angela Fuchs, Hanna Kaduszkiewicz, Mirjam Köhler, Tobias Luck, Edelgard Mösch, Michael Pentzek, Steffi G. Riedel-Heller, Michael Wagner, Siegfried Weyerer, Wolfgang Maier, Hendrik van den Bussche, for the AgeCoDe Study Group

**Affiliations:** 1 Department of Psychiatry, University of Bonn, Bonn, Germany; 2 Institute for Biometrics, Hannover Medical School, Hannover, Germany; 3 Department of Psychiatry, Technical University, Munich, Germany; 4 Central Institute for Mental Health, Mannheim, Germany; 5 Department of General Practice, University Hospital, Düsseldorf, Germany; 6 Department of Primary Medical Care, University Medical Center, Hamburg, Germany; 7 Institute of Social Medicine and Occupational Health, University of Leipzig, Leipzig, Germany; 8 German Center for Neurodegenerative Diseases, Bonn, Germany; Biological Research Center of the Hungarian Academy of Sciences, Hungary

## Abstract

**Background:**

Current approaches for AD prediction are based on biomarkers, which are however of restricted availability in primary care. AD prediction tools for primary care are therefore needed. We present a prediction score based on information that can be obtained in the primary care setting.

**Methodology/Principal Findings:**

We performed a longitudinal cohort study in 3.055 non-demented individuals above 75 years recruited via primary care chart registries (Study on Aging, Cognition and Dementia, AgeCoDe). After the baseline investigation we performed three follow-up investigations at 18 months intervals with incident dementia as the primary outcome.

The best set of predictors was extracted from the baseline variables in one randomly selected half of the sample. This set included age, subjective memory impairment, performance on delayed verbal recall and verbal fluency, on the Mini-Mental-State-Examination, and on an instrumental activities of daily living scale. These variables were aggregated to a prediction score, which achieved a prediction accuracy of 0.84 for AD. The score was applied to the second half of the sample (test cohort). Here, the prediction accuracy was 0.79. With a cut-off of at least 80% sensitivity in the first cohort, 79.6% sensitivity, 66.4% specificity, 14.7% positive predictive value (PPV) and 97.8% negative predictive value of (NPV) for AD were achieved in the test cohort. At a cut-off for a high risk population (5% of individuals with the highest risk score in the first cohort) the PPV for AD was 39.1% (52% for any dementia) in the test cohort.

**Conclusions:**

The prediction score has useful prediction accuracy. It can define individuals (1) sensitively for low cost-low risk interventions, or (2) more specific and with increased PPV for measures of prevention with greater costs or risks. As it is independent of technical aids, it may be used within large scale prevention programs.

## Introduction

The prevalence of dementia is rapidly growing in high-income countries and even more so in countries with low and middle income [1]. As a consequence, the estimated dementia related costs worldwide increased by 34% between 2005 and 2009 up to 422 billion $ [2]. The most effective approach to slow this steep rise in burden and costs is prevention of dementia by early intervention in individuals at increased risk. Recent large efforts in biomarker development have successfully provided a better understanding of pre-dementia brain pathology, particularly of Alzheimer's disease as the most common cause of dementia [3]. These studies are the basis for innovative diagnostic criteria for pre-dementia Alzheimer's Disease [4]. The novel concept of disease identification prior to the onset of clinical dementia has been employed in first clinical trials with compounds that aim at delaying dementia onset [5]. However, current approaches for identification of subjects at pre-dementia disease stages or at high risk for dementia all employ biomarkers, mainly from neuroimaging or cerebrospinal fluid (CSF). These biomarkers require highly specialized settings and sophisticated technology to be reliably assessed.

The vast majority of patients with dementia or pre-dementia conditions worldwide, however, is only seen and treated by non-specialized primary care physicians without access to specific biomarkers. To reach these patients for prevention programs, detection of subjects at increased risk for dementia in primary care is needed. In addition to limited access to biomarkers, the primary care setting is frequently characterized by restricted money and time budget per patient and by an unselected patient population with low disease prevalence [6]. Procedures to identify individuals at risk for dementia in the low prevalence primary care setting with information obtainable under restricted time and cost conditions are lacking.

Dementia prevention strategies may differ in costs and risks. Examples of low cost and low risk strategies would be increased medical attention and counselling on life style. Costs and risks increase, if specific drugs are considered that either modify risk factor or directly act upon the diseases process. Selection of individuals for low risk and low cost interventions should capture most people with the prospective disease even at the expense of sampling subjects, who will not get the disease. In this case prediction should be sensitive, even if high specificity and positive predictive value (PPV) can not be achieved. If the intervention is of higher cost or increased risk, selection should be restricted to those, who will most likely develop the disease at the potential expense of missing some. In this case specificity and PPV should be higher at the expense of sensitivity.

A tool that provides a continuous score rather than a fixed categorical definition can provide different levels of sensitivity, specificity and PPV by using different cut-offs. If the likelihood for a future disease increases with a particular score, specificity and PPV will increase and sensitivity will decrease by raising the cut-off of the score.

In the German Study on Aging, Cognition and Dementia in Primary Care Patients (AgeCoDe) we aimed at creating a quantitative score for primary care physicians to define the risk of an individual for future dementia based on information that can be obtain in the primary care setting in acceptable time and at low costs. We defined two cut-offs. The first was created to sensitively identify subjects at increased risk for dementia. The second cut-off aimed at identifying individuals at high risk for dementia with high specificity and increased PPV. We focused the analyses on Alzheimer's Dementia (AD) as the most common type of dementia.

## Methods

### Ethics statement

The entire study protocol was approved by the local ethical committees of the Universities of Bonn, Hamburg, Duesseldorf, Heidelberg/Mannheim, Leipzig and the Technical University of Munich. Written informed consent was obtained from all participants of this study.

### Participants

The AgeCoDe study is a general practice (GP) registry-based longitudinal study in elderly individuals that aims at identifying predictors of cognitive decline and dementia [7], [8]. The study recruited at six German cities (Bonn, Duesseldorf, Hamburg, Leipzig, Mannheim, Munich). At each site between 19 to 29 GP were connected to the respective study site (138 GP in total). The inclusion criteria for participants were age of 75 years and older, absence of dementia according to GP judgement and at least one contact with the GP within the last 12 months. Exclusion criteria were GP consultations by home visits only, living in a nursing home, severe illness with an anticipated fatal outcome within three months, insufficient German language abilities, deafness or blindness and lack of ability to provide informed consent due to severe mental or sensory impairment or language difficulties.

5102 randomly selected individuals from the GP charts were successfully contacted. 3327 provided informed consent to the GP for participation. Main reasons for not consenting were (more than one answer possible; >10% of cases): no interest in the study (58%), feeling to weak to participate (13%) and lack of time (12%).

The mean age of participants was 80.1 years (SD = 3.6) vs. 80.7 years (SD = 3.8) in those who refused participation (p = 0.019). Within the group of the participants 65.5% were women and 34.5% were men; in the group of non-participants 68.9% were women and 31.1% were men (χ^2^ = 6.028, d.f. = 1, p = 0.014).

The participants were then contacted by the study staff from the respective study centres. All assessments of participants were performed by trained interviewers at the subjects' homes. 85 individuals were excluded after the baseline interview due to the presence of dementia or age below 75 (these were falsely classified as 75 or older in the initial chart selection process). For the present analysis 16 subjects were excluded due to lack of follow-up information on conversion to dementia and 147 were excluded due to conversion to non-AD dementia as we focused on AD only in the present report. These were again included for an exploratory analysis with all dementia cases as reported below. The data-base for the present analyses includes 3055 individuals.

Three follow-up waves with 18 months intervals are the basis for the present analyses. The number of personal interviews was 2634 (86.2%) at follow-up 1, 2338 (76.5%) at follow 2 and 1893 (62.0%) at follow-up 3. The main reasons for not obtaining a personal interview were (1) specific refusal regarding a personal visit due to various reasons (follow-up 1: 63.8%, follow-up 2: 50.5%, follow-up 3: 46%) and (2) death (follow-up 1: 29.7%, follow-up 2: 43.9%, follow-up 3: 38.4%). Informant-based information on those participants without personal interview was obtained from spouses, relatives, caregivers and/or GP on 421 participants at follow-up 1, on 289 at follow-up 2 and on 413 at follow-up 3. The combined follow-up rates (personal interview, informant-based information only) were 100% at follow-up 1, 86.0% at follow-up 2 and 75.5% at follow-up 3. Note that individuals were not followed-up anymore in the case of incident dementia or informant-based information only at one follow-up.

The ApoE genotype was determined in 2938 (96.2%) of participants.

### Assessment procedures

The interviews at baseline at all follow-up assessments included the following procedures.

Subjective memory impairment (SMI) was assessed by the questions: “Do you feel like your memory is becoming worse? ” Possible answers were: “no”; “yes, but this does not worry me” and “yes, this worries me”.

Neuropsychological assessment included the Structured Interview for Diagnosis of Dementia of Alzheimer type, Multi-infarct Dementia and Dementia of other Aetiology according to DSM-IV and ICD-10 (SIDAM) [9]. The SIDAM is specifically designed to diagnose dementia according to the named criteria. It contains (1) a neuropsychological test battery, (2) a 14-item scale for the assessment of activities of daily living (SIDAM-ADL-Scale) and (3) the Hashinski Rosen-Scale [10]. The neuropsychological battery is comprised of 55 items (SIDAM cognitive score, SISCO), including the Mini Mental State Examination (MMSE) [11]. German age- and education-specific norms for the SISCO are published [12].

In addition to the SISCO, the semantic verbal fluency test (naming of animals in 1minute) and the verbal memory test (10-item word list, 3 presentations, delayed recall after 10 minutes) of the neuropsychological battery of the CERAD (Consortium to Establish a Registry for Alzheimer's Disease) were administered [13].

ADL were assessed with the SIDAM ADL scale for definition of dementia (see below). In addition instrumental ADL only were assessed with the Instrumental ADL (IADL) scale [14].

Depressive symptoms were assessed by the 15-item version of the Geriatric Depression Scale (GDS) [15]. Education was classified by the Comparative Analysis of Social Mobility in Industrial Nations (CASMIN) classification system into low, middle and high [16].

The dementia risk factors smoking habits (yes/no) [17] and family history of dementia (yes/no) [18] as well as living status (alone/not alone) were additionally assessed with individual questions. Medical history was obtained from the GPs in all cases.

For those subjects, who could not be interviewed in person at follow-up the Global Deterioration Scale [19]and the subscales “Changes in Performance of Everyday Activities” and “Changes in Habits” of the Blessed Dementia Scale [20] were completed by the interviewer with an informant (spouse, relative, caregiver) and/or with the GP.

### Definition of dementia

Dementia was diagnosed in a consensus conference with the interviewer and an experienced geriatrician or geriatric psychiatrist according to the criteria set of DSM-IV, which is implemented as a diagnostic algorithm in the SIDAM. The algorithm includes cognitive impairment as defined by the SISCO and impairment of activities of daily living as defined by a score of at least two points on the SIDAM-ADL scale. The etiological diagnosis of dementia in AD was established according the NINCDS-ADRDA criteria for probable AD [21]. Vascular dementia diagnosis was guided by the NINDS-AIREN criteria [22], i.e. in case of evidence for cerebrovascular events (Hashinski-Rosen Scale, medical history) and a temporal relationship between the cerebrovascular event and the occurrence of cognitive decline. Mixed dementia was diagnosed in cases of cerebrovascular events without temporal relationship to cognitive decline. For all analyses, mixed dementia and dementia in AD were combined. Dementia diagnosis in subjects who were not personally interviewed was based on the Global Deterioration Scale and the Blessed Dementia Rating subscales. A score of > = 4 on the Global Deterioration Scale was used as the criterion for the dementia diagnosis. In these cases an etiological diagnosis was established, if the information provided was sufficient to judge aetiology according to the criteria named above.

### Statistical analyses

Age, sex, education, the presence of SMI with or without worries, the IADL scale score, the living status, the score on the GDS score, smoking habits (yes/no), family history of dementia in first degree relatives (positive/negative), the verbal fluency score, the verbal delayed recall score and the MMSE score were included as predictor candidates (table 1). With the aim to create a score, variables that are continuous or have multiple categories ware categorized. Age was divided at the mean of the cohort into <80 years and > = 80 years of age. The IADL scale was categorized as impaired or not impaired according to the convention of the scale (impairment: <8 points for women, <5 points for men) [14]. The Geriatric Depression Scale was dichotomized according to the convention of the scale into <6 points (no evidence for depression) and > = 6 points (evidence for depression). The verbal fluency performance was dichotomized into <18 words and > = 18 words in one minute. The delayed recall of the 10-item word list, as the presumably most sensitive measure of prodromal AD, was divided into three categories (0–4 words, 5–6 words, 7–10 words). The MMSE was categorized into <27 points and > = 27 points. The bivariate association of each variable with AD at any follow-up was examined applying χ^2^ test or Linear Trend test for variables with ordered categories, respectively (table 1).

**Table 1:** Description of study sample.

		total cohort(n = 3055)*			first sample (randomly selected from total cohort, n = 1526)		test sample (randomly selected from total cohort, n = 1529)	
		No AD at follow-up	AD at follow-up	p#	No AD at follow-up	AD at follow-up	No AD at follow-up	AD at follow-up
		(n = 2862)	(n = 193)		(n = 1438)	(n = 88)	(n = 1424)	(n = 105)
**Age**	≥ 80 years	1274 (44.5%)	137 (71.0%)	<0.001	628 (43.7%)	64 (72.7%)	646 (45.4%)	73 (69.5%)
**Sex**	MaleFemale	1000 (34.9%)1862 (65.1%)	50 (25.9%)143 (74.1%)	0.011	507 (35.3%)931 (64.7%)	17 (19.3%)71 (80.7%)	493 (34.6%)931 (65.4%)	33 (31.4%)72 (68.6%)
**SMI** 1	no	1248 (43.6)	43 (22.3%)		603 (41.9%)	18 (20.5%)	645 (45.3%)	25 (23.8%)
	yes, without worry	1191 (41.6)	86 (44.6%)		617 (42.9%)	38 (43.2%)	574 (40.3%)	48 (45.7%)
	yes, with worry	423 (14.8)	64 (33.2%)	<0.001	218 (15.2%)	32 (36.4%)	205 (14.4%)	32 (30.5%)
**Verbal fluency**	<18 words	994 (34.7%)	129 (67.2%)	<0.001	492 (34.2%)	60 (69.0%)	502 (35.3%)	69 (65.7%)
**Delayed recall**	7–10 words	1015 (35.7%)	13 (7.0%)		501 (34.9%)	7 (8.4%)	514 (36.4%)	6 (5.8%)
	5–6 words	966 (33.9%)	47 (25.3%)		506 (35.3%)	21 (25.3%)	460 (32.6%)	26 (25.2%)
	0–4 words	866 (30.4%)	126 (67.7%)	<0.001	427 (29.8%)	55 (66.3%)	439 (31.1%)	71 (68.9%)
**MMSE** 2	<27 points	663 (23.2%)	107 (55.4%)	<0.001	344 (23.9%)	56 (63.6%)	319 (22.4%)	51 (48.6%)
**GDS** 3	≥6 points	238 (8.3%)	29 (15.1%)	0.001	121 (8.4%)	14 (15.9%)	117 (8.2%)	15 (14.4%)
**IADL** 4	impaired	226 (7.9%)	37 (19.2%)	<0.001	110 (7.6%)	21 (23.9%)	116 (8.1%)	16 (15.2%)
**Education** 5	low	1758 (61.4)	128 (66.3%)		917 (63.8%)	59 (67.0%)	841 (59.1%)	69 (65.7%)
	middle	794 (27.7%)	46 (23.8%)		368 (25.6%)	22 (25.0%)	426 (29.9%)	24 (22.9%)
	high	310 (10.8%)	19 (9.8%)	0.246	153 (10.6%)	7 (8.0%)	157 (11.0%)	12 (11.4%)
**Living status**	alone	1462 (51.1%)	104 (53.9%)	0.451	737 (51.3%)	52 (59.1%)	725 (50.9%)	52 (49.5%)
**Smoking**	yes	218 (7.6%)	11 (5.7%)	0.327	118 (8.2%)	6 (6.8%)	100 (7.0%)	5 (4.8%)
**Family history**	Positive for dementia	544 (19.0%)	40 (20.7%)	0.557	274 (19.1%)	16 (18.2%)	270 (19.0%)	24 (22.9%)
**ApoE genotype**	ε4 carrier	538 (19.5%)	68 (37.0%)	<0.001	294 (21.4%)	28 (33.3%)	244 (17.7%)	40 (40.0%)

* All variables except ApoE genotype: Number of missing values: 0–22; ApoE genotype: Number of missing values: 117.

# χ^2^ test or Linear Trend test for group comparison.

1 subjective memory impairment,

2 Mini-Mental-Status-Examination,

3 Geriatric Depression Scale,

4 Instrumental Activities of Daily Living Scale,

5 according to the Comparative Analysis of Social Mobility in Industrial Nations (CASMIN) classification system.

The cohort was then split randomly into two samples of equal size using the first as the sample to develop the risk score, and the second as the test sample to assess the predictive accuracy of the score [23], [24].

Multivariate Cox proportional hazard regression was performed to assess the influence of the candidate predictors on the time to onset of AD in the first cohort. A backward stepwise selection of variables based on the Schwarz Bayesian information criterion (BIC) was applied to reduce overfitting [25]–[28]. The BIC penalizes the log likelihood of a model (a measure of its fit) by a factor related to the number of predictor variables in the model (a measure of its complexity) and the number of cases [29]. A reduction of BIC indicates model improvement.

For the calculation of a risk index all predictors of the final model were used. The risk index was calculated as the sum of the respective β coefficients of each factor. To assess the discrimination of the risk index between individuals with and without incidental AD the receiver operating characteristics (ROC), the area under the ROC curve (AUC) and its 95% confidence interval (CI) were calculated. The final model was recalculated with the inclusion of the ApoE4 carrier status to assess the additional effect of ApoE in prediction of AD in this sample.

To create the final scoring system the β coefficients were standardized to an integer score point. The risk score is the sum of these score points.

We defined two cut-offs of the score for different definitions of at-risk groups. The first cut-off should sensitively identify individuals at risk with limited specificity and PPV as a trade-off. For this purpose the cut-off was defined to achieve at least a sensitivity of 80% in the first cohort. The second cut-off should identify a high-risk group with high specificity and increased PPV. This was achieved by defining the upper decile (10%) of the risk score as the risk group only. The cumulative hazard rates for the respective risk groups were calculated using the Kaplan-Meier method [30]. Exploratively, we calculated the PPV for the top 5% of the risk score for AD and for any dementia by including the additional cases with any dementia at follow-up.

For validation, the predictive accuracy for both the risk index and the simplified risk score were assessed in the test sample. In addition, sensitivity, specificity, PPV and NPV for the cut-offs were determined in the test sample and positive and negative likelihood ratios (LR^+^, LR^−^) for both cohorts.

## Results

The baseline characteristics of the participants are listed in table 1. From the 3055 participants, 193 (6.32%) developed AD during follow-up. The mean follow-up time per individual was 3.81 years (maximum: 6.14 years).

### Selection of predictive factors

All 12 factors were included in the multivariate Cox proportional hazard model based and applied to the search sample. The stepwise selection of variables based on the BIC revealed improvement of the model after removing the GDS score (−4.42), smoking status (−4.40), family history of dementia (−4.30), living status (−4.22), education (−7.02) and sex (−1.27). Removing IADL impairment worsened the model as shown by in increased BIC (+1.80). The order of removing variables was determined by their Wald χ^2^. The final model included the predictors age, presence of SMI with and without worry, IADL score, verbal fluency score, delayed recall score and MMSE score.

The estimated β coefficients, the Hazard Risk ratios and the 95% confidence intervals are shown in table 2.

**Table 2:** Cox regression models for Alzheimer dementia risk (first cohort, n = 1526).

		full model				final model				
		β coefficient	p	HR^1^	95% CI	β coefficient	p	HR	95% CI	Score
Age	75–79 years	0		1		0		1		0
	≥80 years	0.959	0.0002	2.610	1.566–4.349	1.015	<0.0001	2.758	1.671–4.555	3
Sex	male	0		1						
	female	0.605	0.0598	1.831	0.975–3.437					
SMI^2^	no	0		1		0		1		0
	yes, without worry	0.622	0.0358	1.863	1.042–3.331	0.630	0.0331	1.876	1.052–3.347	2
	yes, with worry	1.256	<0.0001	3.512	1.898–6.499	1.299	<0.0001	3.662	2.001–6.702	4
Verbal fluency	≥18	0		1		0		1		0
	<18	1.057	<0.0001	2.877	1.746–4.740	1.084	<0.0001	2.956	1.809–4.830	4
Delayed recall	7–10	0		1		0		1		0
	5–6	0.641	0.1516	1.898	0.791–4.555	0.598	0.1780	1.818	0.762–4.338	2
	0–4	1.415	0.0009	4.117	1.791–9.465	1.312	0.0018	3.712	1.630–8.452	4
MMSE^3^	≥27	0		1		0		1		0
	<27	1.107	<0.0001	3.024	1.877–4.873	1.097	<0.0001	2.996	1.872–4.795	4
GDS^4^	<6	0		1						
	≥6	0.018	0.9550	1.018	0.540–1.922					
IADL^5^	unimpaired	0		1		0		1		0
	impaired	0.581	0.0444	1.789	1.015–3.153	0.707	0.0079	2.028	1.204–3.415	2
Education^6^	high	0		1						
	middle	0.270	0.3247	1.311	0.765–2.245					
	low	0.489	0.2380	1.630	0.724–3.671					
Living status	not alone	0		1						
	alone	−0.118	0.6429	0.889	0.540–1.462					
Smoking	no	0		1						
	yes	−0.053	0.9060	0.948	0.394–2.282					
Family history	Negative for dementia	0		1						
	Positive for dementia	−0.099	0.7320	0.905	0.513–1.599					

1 Hazard Ratio,

2 subjective memory impairment,

3 Mini-Mental-Status-Examination,

4 Geriatric Depression Scale,

5 Instrumental Activities of Daily Living Scale,

6 according to the Comparative Analysis of Social Mobility in Industrial Nations (CASMIN) classification system.

Using these variables, the area under the ROC curve (AUC) of the risk index was 0.84 (95% CI: 0.80–0.88) in the first cohort.

In a second analysis in the first cohort, the Apoe4 status (carrier/non-carrier) was included as a predictor. The estimated β coefficients, the Hazard Risk ratios and the 95% confidence intervals were similar as in the model without ApoE4 carrier status without any change in significance (data not shown). The added hazard ratio of the ApoE4 status itself was not significant. The AUC of the corresponding risk index of the model with ApoE4 carrier status was 0.85 (95% CI: 0.80–0.89).

### Risk score for AD

**Figure 1:**
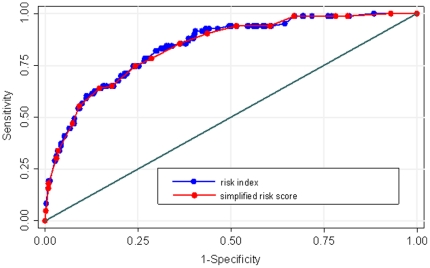
Receiver operating characteristics (ROC) of the risk index and the simplified risk score in the first cohort.

From the final model a simplified risk score was derived by multiplying the β coefficients with 10/3. The multiplication with 10/3 was chosen because most of the β coefficients were close to divisible by 3, thus rounding errors were kept small. Scoring points are presented in table 2. For an individual, the risk score is the sum of the score points of each predictor (maximum 21 points). The AUC for the corresponding ROC curve was 0.84 (95% CI: 0.80–0.88). There was no significant difference between the AUC of original risk index and the simplified risk score (p = 0.063, see figure 1).

### Validation in the test cohort

**Figure 2:**
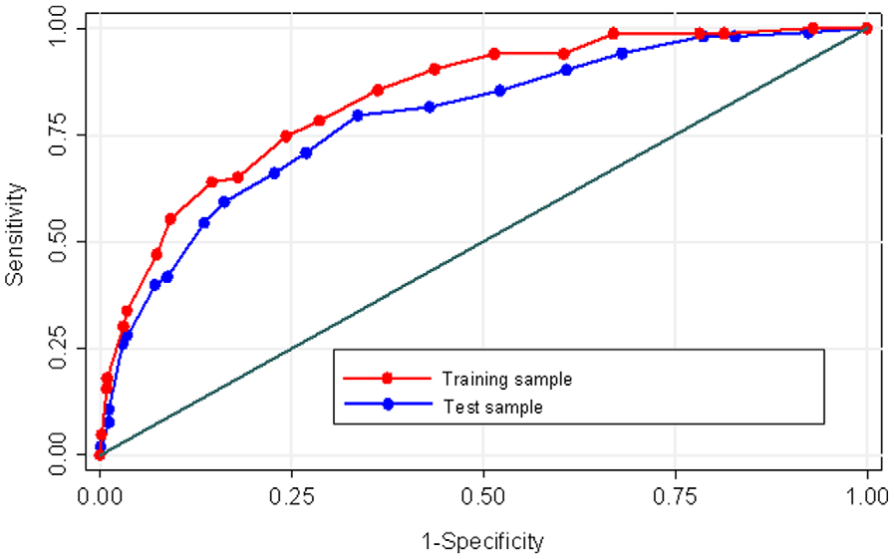
Receiver operating characteristics (ROC) of the risk score in the first cohort and in the test cohort.

Applying the risk score of the model to the test sample revealed an AUC of 0.79 (95% CI: 0.74–0.84), which was not significantly different from the AUC of the first sample (p = 0.13). Both curves are depicted in figure 2.

### Prediction of AD

The risk score cut-off had to be positioned at > = 9 points to achieve the requirement of at least 80% sensitivity in the first cohort. To define the high-risk group, the risk score was dichotomized into the upper decile and the rest of participants in the first cohort (> = 15 points).

At a cut-off of > = 9 points the score achieved a sensitivity of 85.5%, a specificity of 63.8%, a PPV of 12.0% and a NPV of 98.7% for the prediction of AD in the first cohort (LR^+^ = 2.36, LR^−^ = 0.23). In the test cohort a sensitivity of 79.6%, a specificity of 66.4% and PPV of 14.7% and a NPV of 97.8% was achieved (LR^+^ = 2.37, LR^−^ = 0.31).

The decile division reached a sensitivity of 28.9%, a specificity of 92.8%, a PPV of 28.9% and a NPV of 95.5% for the prediction of AD in the first cohort (LR^+^ = 5.53, LR^−^ = 0.65). In the test cohort a sensitivity of 47.0%, a specificity of 92.6% and PPV of 26.9% and a NPV of 96.8% was achieved (LR^+^ = 6.35, LR^−^ = 0.57).

**Figure 3:**
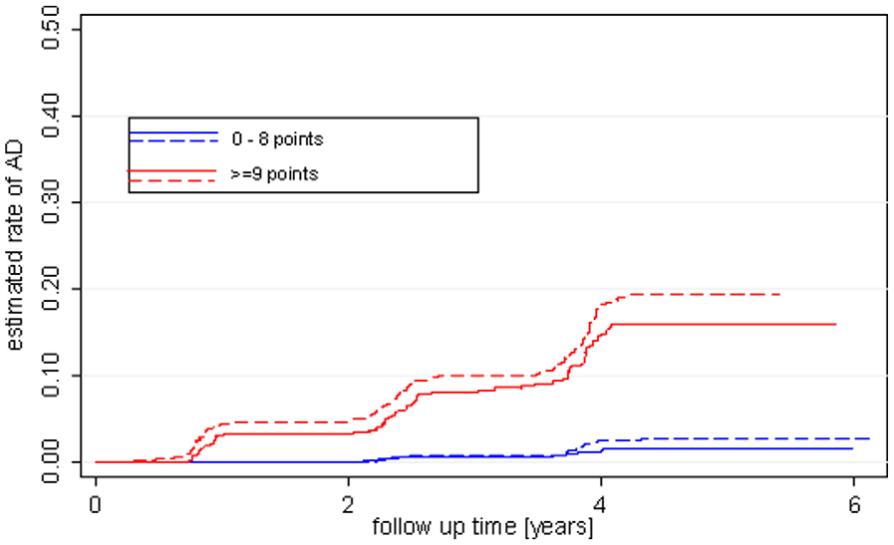
AD-free survival in the first cohort and the test cohort by the criterion of at least 80% sensitivity in the first cohort (0–9 and > = 10 points).

**Figure 4:**
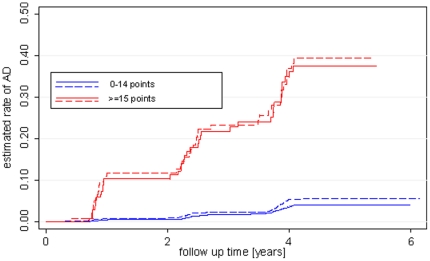
AD-free survival in the first cohort and the test cohort divided into the top 10% on the risk score and the rest of the cohorts (0–14 and > = 15 points).

The rates of progression to dementia for both cut-offs are listed table 3. Figures 3 and 4 display the respective survival curves.

**Table 3:** Rate of progression to AD by risk score.

		Training cohort			Test cohort		
	Baseline risk score	Number of AD cases at follow-up	Mean time to incident AD	95% CI	Number of AD cases at follow-up	Mean time to incident AD	95% CI
***Group definition 1*** a							
	0–8	12 (1.3%)	5.9	5.9–5.9	21 (2.2%)	6.1	6.1–6.1
	≥9	71 (12.0%)	5.4	5.3–5.5	82 (14.7%)	4.9	4.8–4.9
	Total	83 (5.5%)	5.8	5.7–5.8	103 (6.8%)	5.9	5.8–5.9
***Group definition 2*** b							
	0–14	44 (3.2%)	5.9	5.8–5.9	62 (4.5%)	5.9	5.9–6.0
	≥15	39 (26.9%)	4.3	4.1–4.6	41 (28.9%)	4.3	3.9–4.6
	Total	83 (5.5%)	5.8	5.7–5.8	103 (6.8%)	5.9	5.8–5.9

a The cut-off of the risk score was defined as to achieve a sensitivity of at least 80% in the first cohort.

b The cut-off was defined to separate the 10% individuals with the highest risk score from the rest in the first cohort and to define them as a high risk group. AD = Alzheimer's dementia.

## Discussion

The aim of this study was the creation of a score for the identification of individuals at risk for AD in elderly primary care patients. All variables of this score should be easily obtainable in the primary care setting in acceptable time and at low cost. The interpretation should be straight forward based on cut-offs. We identified significant predictors for AD out of a larger set of variables, created the score in a randomly selected first cohort and validated the score in second half of the sample (test cohort).

The prediction accuracy (AUC) of the score was 0.84 in the first cohort and 0.79 in the test cohort. We defined two cut-offs, one with high sensitivity of >80% in the first cohort and limited specificity and PPV and one with high specificity and increased PPV. The first cut-off (> = 9 points) achieved a sensitivity of 79.6%, a PPV of 14.7% and a NPV of 97.8% in the test cohort. This cut-off is sensitive and potentially over inclusive. It can be applied, if the consequence of being at risk according to this cut-off is of low risk and low cost for the individual. Subjects at risk according to this definition may receive intensified counselling regarding modifiable risk factors related to lifestyle and may receive increased clinical attention to identify first signs of dementia.

The second-cut off (>/ = 15) points reached a specificity of 92.6%, a PPV of 26.9% and a NPV of 96.8% in the test cohort. Those individuals scoring above this cut-off are at high risk with every fourth subject converting to AD within the observational period. Individuals in the high risk group according to this definition could be subject to intensified pharmacological and non-pharmacological prevention programs that might be developed in the future [4].

Both cut-offs achieved NPV above 95% indicating that low scoring on the risk score is associated with very low risk for AD at follow-up.

It needs to be stressed at this point that in low prevalence populations as in primary care the PPV tends to be low and the NPV tends to be high compared with high prevalence populations that characterize specialized settings. This is also the case for several other medical conditions that occur in primary care, such as depression [31]. This effect is caused by the unselected nature of low prevalence populations, which includes individuals, who fulfil at-risk criteria due to any reasons and not only due to the prodromal disease of interest (AD in the case of this study). These individuals are a priori excluded from the highly selected population of specialized settings. As such, the measures reported here cannot be directly compared to high PPV obtained in biomarker studies in high prevalence cohorts from specialist settings.

Other prediction scores for dementia have been published. In one study a risk score for dementia prediction over the course of 20 years was generated from a cohort with an age at baseline of 50 years on average. The authors identified age, education, sex, systolic blood pressure, body mass index, total cholesterol, physical activity and the ApoE4 status as components of the score. They reported a prediction accuracy (AUC) of 0.77 with a PPV of 9% and a NVP of 98% [32]. This score highlights the relevance of mid-life risk factors for dementia. However, due to the long prediction period of 20 years and midlife age at baseline, it is clinically not useful for dementia risk assessment in elderly primary care patients.

Another score for prediction of dementia was derived from a population-based cohort with a mean baseline age of 76 years and an observation period of 6 years. The score included the predictors age, cognitive test performance, body mass index, ApoE4 status, white matter lesions on MRI or ventricular enlargement, internal carotid thickening, history of by-pass surgery, slow physical performance and lack of alcohol consumption [33]. The accuracy of prediction (AUC) was 0.81. The PPV was 57% for individuals scoring in the top 5% of the prediction score for any dementia. In our analysis, we defined the top scoring 10% on the risk score as the high-risk group. Narrowing the high-risk definition to 5% in our data would have increased the PPV to 39.1% for AD. Employing any dementia exploratively as an outcome, the PPV was 52.0% for the top scoring 5% on the risk score in the test cohort of our study. However, a group size of only 5% might be of limited utility in clinical practice for the definition of individuals that may receive specific programs or treatments. Any dementia as the prediction target instead of only AD is unspecific and limits the application of the risk score, if actions specifically tailored for pre-dementia Alzheimer's disease are considered.

Importantly, the AUC of our data and those of the other prediction scores are in a similar range indicating similar performance. In contrary to the score reported by Barnes et al., our score did not include components derived from technical investigations such as MRI or ultrasound [33].

However, we included clinical information that was not included in the other scores. In our data, SMI significantly predicted AD. This is in agreement with the majority of longitudinal studies that found an association of SMI with future cognitive decline and dementia [34], [35]. Importantly, characteristics of SMI that induce worry in individuals are associated with greater risk than SMI that does not cause worry. It needs to be stressed, however, that not all individuals report SMI in the prodromal phase of AD [e.g. 36].

In our data impairment in IADL contributes to the prediction score. Note, that patients with clearly impaired ADL fulfilling dementia criteria were excluded at baseline. Impairment in IADL has been identified as an important predictor of dementia in subjects with mild cognitive impairment (MCI) in other epidemiological studies [37], [38]. The inclusion of IADL impairment highlights the relevance of functional impairment in addition to purely cognitive impairment in the prediction of AD.

In our data delayed episodic memory performance, as measured by word list recall, contributed to the risk score. This is in agreement with current concepts of AD proposing episodic memory impairment as the cardinal feature of cognitive decline in early AD [4]. Verbal fluency performance and the MMSE score as a measure of global cognitive function also contributed to the prediction score. This is in agreement with studies showing that MCI subjects with impairment in more cognitive domains than just episodic memory (multi domain amnestic MCI) are at particular high risk for dementia [39], [40]. In other longitudinal cohorts either specific memory tests or global tests of cognition alone achieved reasonable dementia prediction accuracy [41], [42], [43]. Direct comparison of these studies with our data is limited by differences in setting, subjects, instruments and other factors. In our model, however, we found improvement of prediction by including those clinical variables listed above in addition to cognitive tests.

In this study risk modifying factors for AD such as sex, family history of dementia, depressive symptoms, education and smoking [44], [45] did not contribute independently to the prediction of AD. This suggest that prodromal symptoms of dementia such as subjective decline, cognitive impairment and mild impairment of function contribute to prediction, whereas the independent effects of pure risk factors are minor in predicting dementia in elderly subjects over a limited number of years.

In agreement, the ApoE4 status also did not contribute independently to risk prediction of AD and did not increase the performance of the risk index. This suggests that determination of the ApoE genotype in not necessarily required for risk assessment in the primary care patient population above 75 years of age.

This study has limitations. The inclusion age was 75–90 years in order to define risk for dementia in high age individuals. Consequently, the prediction score cannot be directly applied to younger age groups.

The observational period per participants was 3.8 years on average (maximum 6.14 years). Thus, our data reflect prediction in a rather short time frame. A longer follow-up frame with more incidental AD case would have provided greater accuracy of prediction estimates.

The diagnosis of AD was based on interview and test material. It did not include brain imaging. However, it is unlikely that the increased validity of the etiological diagnosis achieved by the inclusion of brain imaging would weaken the performance of the risk score as the score empirically reflects the conceptual components of early symptom manifestation of AD. It can be speculated that the prediction of AD would have been even more accurate, if brain imaging would have been included to establish the diagnosis.

We restricted the primary analyses to AD as the most common type of dementia. The strategy was chosen, because AD is conceptually well defined and most knowledge on prevention of dementia refers specifically to AD.

In our study, we derived the score from one half of the cohort and tested it in the other half. However, the performance of the score needs to be replicated in independent samples from different language and socioeconomic backgrounds to test its validity for widespread use.

In conclusion, we identified a set of predictors and we created a risk score for AD in elderly primary care patients. The relevant components of the score are (1) the report on memory impairment by the individual on active inquiry (SMI) plus the quality of this subjective impairment (worrisome/not worrisome), (2) performance on a global cognitive test (MMSE), and on a more specific tests of episodic verbal memory (10-item word list learning) and of verbal fluency (e.g. naming of animals in one minute), (3) performance of IADL, and (4) age. All required information is obtainable in daily practice without any major technical effort. In contrast to a categorical risk definition, such as MCI, the score can serve different purposes by varying the cut-off. The score can guide primary care physicians' decision in individual patients on actions such as increased clinical attention, counselling as well as initiation of measures for prevention and for early diagnosis.
